# Growing risk of child labor in the Middle East and North Africa amid cascading crises

**DOI:** 10.3389/fpubh.2025.1502687

**Published:** 2025-04-01

**Authors:** Rima R. Habib, Reem S. Katrib, Diana Mikati, Marian Abouzeid

**Affiliations:** ^1^Faculty of Health Sciences, American University of Beirut, Beirut, Lebanon; ^2^Centre for Humanitarian Leadership, Deakin University, Geelong, VIC, Australia; ^3^Alfred Deakin Institute, Deakin University, Geelong, VIC, Australia

**Keywords:** child labor, Sustainable Development Goals (SDGs), Target 8.7, risk factors, armed conflict, Middle East and North Africa (MENA)

## Introduction

Child labor is a global health problem and is associated with a range of adverse health outcomes (1). Globally, 160 million children between 5 and 17 years are engaged in child labor, of whom 79 million are performing hazardous work (2). By definition, hazardous work is likely to harm the health, safety or morals of children and may result in death, disability, or longstanding physical or psychological damage (3). Child labor is intertwined with development: prevalence is inversely proportional to human development indices, and is three times higher in fragile countries than the global average (4). Prevalence of child labor also varies markedly by geographic region; although the period 2016–2020 saw a decline in the absolute numbers in several regions including Asia, the Pacific and Latin America and the Caribbean, other regions such as Sub-Saharan Africa experienced massive increases and globally, the absolute number of children in child labor increased by over 8 million (5).

Even prior to the COVID-19 pandemic, at the existing pace of change and without accelerated action, it was estimated that almost 140 million children globally would still be in child labor by 2025 (4). The pandemic shifted the landscape, with further estimates that worldwide, an additional 8.9 million children were at risk of being forced into labor by the end of 2022 due to rising poverty driven by the pandemic, and modeling suggesting that in the absence of social protection supports, this figure could rise by a staggering 46 million (4).

Concerningly, comprehensive and up-to-date prevalence data is scarce or patchy for some geographic regions; country-specific monitoring mechanisms on child labor are lacking in some settings, (6) and additionally, there are disparate prevalence estimates from different sources.

There have been considerable global efforts directed toward addressing child labor. Target 8.7 of the Sustainable Development Goals (SDGs) seeks to end child labor in all its forms by 2025 (7). Recent years have seen several landmark global events and novel initiatives, including the 2016 launch of Alliance 8.7 (8), establishment of the International Labor Organization's Research to Action (RTA) project (9), declaration of 2021 as the International Year for the Elimination of Child Labor (10), and the 2022 movement of the “Durban Call to Action on the Elimination of Child Labor.” (11). Despite these efforts, the world has failed to meet Target 8.7. New approaches are urgently needed if this tragic problem and major social determinant of health is to be effectively addressed.

## Constellation of risk factors for child labor in the MENA region

Numerous factors influence progress toward reducing child labor, reflecting broader development progress. These vary by geographic region, and so too the effects of global crises on developmental progress, poverty and child labor risks will be differentially felt, likely magnified in settings experiencing other shocks and cascading crises, including humanitarian crisis-affected settings and fragile states where social infrastructures are compromised and the ability to absorb further shocks is limited. The MENA region, which spans 20 countries including Algeria, Bahrain, Djibouti, Egypt, Iran, Iraq, Jordan, Kuwait, Lebanon, Libya, Morocco, Oman, Qatar, Saudi Arabia, State of Palestine, Sudan, Syrian Arab Republic, Tunisia, United Arab Emirates, and Yemen (12), is experiencing numerous humanitarian crises and has the largest humanitarian funding requirements of all regions globally (13). It is reported that in early 2020, an estimated 6.5% of children in MENA aged between 5 and 17 years, representing a staggering 7.2 million people, were engaged in child labor (4). This is likely to be a gross underestimate, given the constraints and limitations of the region's child labor surveillance mechanisms and the likely surveillance disruptions resulting from crises in and the fragility of the region. Recent events in the region also render this figure outdated.

The region has a constellation of risk factors for child labor, including due to immense political instability, armed conflicts, natural hazards (14) and poor protection measures in many settings, amplified by the impacts of climate change, the lingering effects of the pandemic, and other global crises.

### COVID-19 and child labor risks across the region

The COVID-19 pandemic highlighted the harsh reality of absent social protection in many MENA countries, leading to an increased vulnerability of children and particularly those living in precarity (15). As elsewhere, the pandemic and response measures had pronounced economic effects in MENA, with adverse consequences on children's health, wellbeing, and development (16). Over the course of the pandemic, indicators for all forms of child labor worsened, attributed to factors including poor social protection measures, lack of universal education access, and an exploitative informal sector (15). It is reported that the primary cause for the increase in child labor was the economic impact of the COVID-19 pandemic, which caused high unemployment, greater poverty, reduced remittances, and triggered migration (15), with children pushed to work to support their families to meet basic needs (14). The pandemic also aggravated the domestic work burden, with increasing reliance on children, especially girls, to support with domestic chores and caring responsibilities (4).

There are also longer-term potential impacts of COVID-19 on vulnerability and child labor risk. Long COVID, which threatens to generate a considerable global burden of disability (17), may have profound flow-on effects for child labor risk. For example, workforce shortages may push children into labor in some settings; additionally, if parents and caregivers are unable to work due to the disabling sequelae, children may be forced into the labor market as a coping mechanism to support families.

The indirect impacts of the pandemic on child labor risk could also persist across the lifespan as a result of poor employment opportunities, the development of chronic health issues among working children, (18) and the impact of education disruption and early school dropout on life trajectories.

### Massive economic collapse across the region

A 1% increase in poverty reportedly leads to at least a 0.7% increase in child labor (19). Several countries in the MENA region are currently experiencing unprecedented recession and economic hardship. For example, Lebanon is in the grips of a massive economic collapse that the World Bank has described as one of the most severe globally since the mid-19th century. This crisis has had a range of devastating impacts, including rising rates of child labor and abuse (20). Such dire economic contexts may severely exacerbate vulnerabilities and expose children to hardship, pushing them into the labor market.

### Armed conflicts destroy social and protection infrastructures

In many countries across the region, protracted conflicts and humanitarian crises have had profound and devastating impacts on the safety and wellbeing of children and placed a generation at risk. In addition to the devasting direct impacts of war on children's lives, health and wellbeing, in conflict settings child protection is also compromised through numerous pathways and risk factors of child labor are aggravated. Amongst other impacts, conflicts often provide the context for school closures and the military use of schools. Arguably among the worst and most abusive forms of work, in some conflict settings in the region, children have been pushed to engage as child soldiers, with schools sometimes used as a recruitment ground (21).

In both conflict-affected and refugee-hosting settings, displacement, parental death, increased food insecurity, and extreme poverty have been associated with an increased prevalence of child labor (22). Formal employment opportunities for refugees are often limited due to restrictions on their residence status and access to the labor market; this drives children into the workforce, whereby they have reduced access to education, endure very long working hours, and earn low wages as a result of their precarious situations (23). One such example is that of Syrian refugee children in Lebanon. The refugee population in Lebanon struggles to obtain employment permits, with 95% of those employed reporting a lack of valid work permits (23). This can result in negative coping mechanisms by refugee families, including withdrawal of children from education, that can increase the likelihood of being pushed into labor (24). As there are also widespread limitations to the legal framework to protect children, combatting child labor for all children becomes increasingly difficult (24).

Wars and armed conflicts also disrupt social infrastructures that help prevent child labor. For example, effective and functioning civil registration and vital statistics (CRVS) systems are integral to child protection, including legal identity and proof of age documentation being essential to prevent child labor (25). In the MENA, as of 2019 it was estimated that 4 million children under 5 years had not had their births registered, and 7 million did not have a birth certificate and do not officially exist (25). Although the region overall has comparatively high levels of birth registration, it is reported that there has been no progress toward universal registration since 2000 (25). There is also marked variation between countries—whilst some have achieved or are close to universal registration and have well-functioning CRVS systems, others such as Yemen have low birth registration rates (25). The state of CRVS infrastructures, quality assurance processes, and the barriers to improved functions vary (25). Additionally, armed conflicts can result in profound disruption or collapse of existing CRVS infrastructures, and the populations displaced by wars and armed conflicts also face numerous barriers to birth registration (25).

### Climate change and natural hazards exacerbate the cycle of precarity

The MENA region is highly susceptible to the impacts of climate change. The effects of climate change have been shown to influence the risk of child labor and the conditions under which work is undertaken, often resulting from increased socioeconomic vulnerability and food insecurity from climate-related shocks (26). Separation from their families or becoming orphaned place children at risk of child labor; natural hazards tend to increase this risk (26).

Climate change and conflict are also intertwined. With the MENA region among the most vulnerable to the impacts of climate change, climate change will have a devastating toll on the region's water supplies and food production systems, and subsequently has potential to create breeding grounds for violence (27).

Amongst a complex constellation of other factors, for example, Syria's civil war has been partially attributed to the five-year drought in the country which began in 2007 (28). Burdened by population growth, the drought resulted in unprecedented poverty and triggered migration to Syria's major cities. Water scarcity fuels tensions, disrupting the infrastructures that help prevent child labor and pushing families into negative coping mechanisms like child labor.

Natural hazards and the politics of aid also increase vulnerability and child labor risk, as is reported for example following the devastating earthquakes in Syria in early 2022 (29), and the limitations of the humanitarian response including international community mobilization, which further placed children at risk.

## Discussion

Over recent years, there have been a range of initiatives to address child labor in several settings across the region, including in Egypt, Lebanon, Syria, Yemen, Morocco and Jordan (30). However, the world has failed to meet its target of ending child labor in all its forms by 2025. Indeed, the MENA region today is at risk of soaring child labor prevalence due to a unique synergistic mix of risk factors stemming from both local and global crises. A new region-specific and locally-led approach to address child labor is urgently needed, alongside renewed global efforts. Initiatives that strengthen local structures and systems weakened by crises are essential to better protect children socially and legally from child labor and its harmful effects. These must consider and account for the unique socioecological, political, economic, and cultural contexts of children and of the region. For instance, education policies and interventions targeting children, families, schools, and teachers, and communities can help decrease children's participation in economic activities (31). Migrant-inclusive welfare systems and comprehensive crisis management approaches could help protect and promote the rights, health, and wellbeing of vulnerable children (22). Additionally, climate action plans should prioritize the needs of children and child labor elimination strategies, such as through increased financial commitments to support climate migrant families and effective migration policies. Enhancing cross-sectoral cooperation and establishing a tangible and realistic strategic action plan that is multisectoral, with partnerships between state actors, international organizations, regional, national and grassroots level organizations, academic institutions, diaspora and the private sector, coupled with a rigorous monitoring system for accountability and transparency would be a step in the right direction. Standardized, transparent and periodic reporting of prevalence estimates is imperative to monitor progress and inform targeted action, particularly given variable prevalence data from across the region.

Although fundamentally political, addressing the root causes of child labor risks across the region is also crucial. The “Durban Call to Action on the Elimination of Child Labor” (11) has set the right commitments to address child labor including peacekeeping and peacebuilding, poverty reduction, and addressing inequalities; however, it does not present a tangible trajectory to achieve these commitments.

Having failed to meet Target 8.7, it is now more important than ever to take stock and for renewed global ambitions alongside strong and substantial local and regional action. This is particularly pertinent given the renewed landscape in many parts of the region that have endured brutal conflicts recently, including Palestine, Lebanon and Syria.

Children in the MENA are subject to a range of threats and their protection must be at the forefront of all national, regional and global developmental efforts. Given the constellation of precarious social determinants and drivers of child labor in MENA, failure to address the risk factors for increased child labor in this region will further increase the likelihood of a generation being left behind.
